# VANISH: a challenge for current sepsis guidelines!

**DOI:** 10.1186/s13054-016-1515-y

**Published:** 2016-10-31

**Authors:** Sebastian Rehberg, Matthias Gründling, Christian Ertmer

**Affiliations:** 1 Department of Anesthesiology, Anesthesia, Intensive Care, Emergency and Pain Medicine, University Hospital of Greifswald, Ferdinand-Sauerbruch-Str., 17475 Greifswald, Germany; 2 Department of Anaesthesiology, Intensive Care and Pain Medicine, University Hospital of Muenster, Albert-Schweitzer-Campus 1, building A1, 48149 Muenster, Germany

Haemodynamic therapy in septic shock with non-adrenergic compounds such as the vasopressin-receptor agonist arginine vasopressin (AVP) [1], the calcium-sensitizer levosimendan [2], or beta-blockers [3] is gaining more and more attention since the potential negative effects of catecholamines in shock are well recognized [4]. The VANISH trial focused on the effects of AVP versus norepinephrine on renal failure (as the primary outcome) and mortality rates at 28 days and serious adverse events (as secondary outcomes) [1]. Although there might have been a potential benefit on kidney-failure-free days, overall the trial revealed no statistically significant differences regarding these outcomes. Thus, just another negative, randomised controlled trial in sepsis? Not at all!

Although not pointed out or discussed by the authors, the therapeutic regime used for AVP itself represents a major breakthrough for the use of non-adrenergic vasopressors as it strongly challenges current recommendations for the treatment of severe sepsis and septic shock [5] in multiple ways. First, the maximum dose was doubled (0.03 versus 0.06 U/min), suggesting a shift from the original “hormone replacement” therapy to a haemodynamic-guided vasopressor treatment. Second, AVP was started within 6 h of diagnosis, i.e. early in the time course of septic shock. This regime challenges the common approach to add AVP only if standard vasopressors become ineffective. Third, and probably the most important advance, was to use AVP as a first-line therapy. Accordingly, AVP was tested as a replacement for the “gold standard” norepinephrine (not as a supplement) in a large, multicentre, randomised controlled trial for the first time. This clearly contradicts current recommendations suggesting that “vasopressin is not recommended as the single initial vasopressor for treatment of sepsis-induced hypotension” [5].

Notably, the present results reveal that this therapeutic approach with the non-adrenergic vasopressor AVP is not only feasible and safe, but also is as effective as the standard treatment with norepinephrine; not to forget the potential beneficial effects on kidney failure. In conclusion, the authors have affirmed a new therapeutic approach for the haemodynamic management of septic shock with the highest evidence level. Following this fundamental step, future studies are warranted for verification, to determine long-term outcomes, and to identify patient populations who benefit the most from this non-adrenergic therapeutic regime.

## Abbreviation

AVP: Arginine vasopressin
